# A social network graph partitioning algorithm based on double deep Q-Network

**DOI:** 10.1038/s41598-025-16768-x

**Published:** 2025-10-02

**Authors:** Jie Cao, Haoxiang Wang, Jingru Jiao, Kekun Hu, Ping Qi

**Affiliations:** 1 School of Mathematics and Computer Science, TongLing University, TongLing, 244000 China; 2 The College of Software, Zhengzhou University of Light Industry, Zhengzhou, 450002 China; 3 IEIT SYSTEMS Co., Ltd, Jinan, 250101 China

**Keywords:** Social networks, Graph partitioning, Graph convolutional neural network, double deep Q-Network, Computer science, Software

## Abstract

With the rapid expansion of social networks, efficiently mining and analyzing massive graph data has become a fundamental challenge in social network research. Graph partitioning plays a pivotal role in enhancing the performance of such analyses. However, conventional graph partitioning methods predominantly rely on local structural information and often overlook the rich attribute information associated with vertices in social network graphs. To overcome this limitation, this paper introduces GP-DQN (Graph Partitioning via Double Deep Q-Network), a large-scale graph partitioning algorithm that jointly considers structural correlations, attribute disparities among user vertices, and partition load balancing. GP-DQN encodes partition load metrics and vertex attributes into vector representations and employs a Graph Convolutional Network (GCN) to aggregate both vertex features and neighborhood structures, thereby improving the accuracy and scalability of the partitioning process. A tailored reward function is designed to guide partitioning actions, where a Double Deep Q-Network (DDQN) predicts the expected partitioning rewards based on GCN-extracted features for assigning each vertex to different partitions. The partitioning strategy is iteratively optimized using both immediate and expected rewards, ultimately achieving balanced load distribution while minimizing the number of edge cuts. Experimental results demonstrate that GP-DQN produces well-balanced partitions with significantly fewer edge cuts, leading to enhanced computational efficiency within each partition.

## Introduction

In recent years, with the rapid development of technologies such as mobile internet, the internet of things, social networks, and big data processing, various industries have begun to generate vast and diverse datasets continuously^1^. In most datasets, relationships often exist between data items, which can be modeled using graphs. Vertices in the graph represent data items, while edges represent relationships between them. As social networks scale up, the volume of data they produce grows exponentially. WeChat, for example, has 1.1 billion daily active users, with 45 billion messages sent daily, 780 million users browsing their moments, and 120 million users posting updates, that include 670 million photos and 100 million short videos. Facebook, the world’s largest social network, currently has about 1 billion users and tens of billions of relationship links, posing significant challenges to the timeliness of data analysis, data storage, and service delivery in social network-based applications.

Social networks are typically represented as attributed graphs, where both vertices and edges carry attributes. Such graphs have complex community structures, where densely connected user vertices form communities with tighter internal connections and sparser inter-community links^2^. Moreover, social network graphs follow a pronounced power-law distribution: a few vertices have extremely high connectivity, while most vertices have few connections^3^. Leveraging community structures and vertex attributes can enhance social network analysis, value extraction, and applications like personalized recommendation and targeted advertising. The power-law property also helps more accurate anomaly detection in social network graphs and improves the efficiency of graph algorithms by capitalizing on the skewed connectivity pattern. Early social networks were sufficiently small to be stored and processed on a single machine. As their scale expanded, however, a single processing node could no longer efficiently handle large-scale social network graphs. It has driven the development of distributed graph processing models, which partition large graphs across multiple computing nodes to enable parallel processing^4^. For instance, Ma et al.^5^ developed a distributed graph neural network that uses parallel strategies to reduce data transmission and redundant computations, enhancing the performance of processing large social network graphs. Weng et al.^6^ proposed a large-scale graph kernel decomposition algorithm, executing kernel decomposition based on global activation and hierarchical peeling in each superstep of the Bulk Synchronous Parallel (BSP) model, and optimizing performance through local priority and message pruning strategies. Graph partitioning is a foundational technique in distributed graph processing (also known as graph computation), and it plays a crucial role in enabling efficient graph storage, query execution, processing, and data mining^7^. Effective partitioning distributes graph data across computing nodes to balance storage loads and improve resource utilization. When queries involve only local partition data, searching within the partition significantly reduces the search scope and speeds up query response. Many graph mining algorithms rely on local information propagation or iterative updates; suitable partitioning helps these algorithms converge faster and reduces the need for global synchronization. Graph partitioning involves dividing a large graph into smaller subgraphs to facilitate distributed computing or optimize storage. It mainly falls into two categories based on the partitioned object: vertex partitioning and edge partitioning. Vertex partitioning assigns vertices to different partitions, with each partition containing a subset of vertices and their incident edges. An edge is cut if its two endpoints are in different partitions. The goal is to minimize the number of cut edges while balancing the vertex count across partitions to reduce inter-partition communication overhead. Graph processing systems that adopt vertex partitioning include PowerGraph and Pregel^8^. In contrast, edge partitioning assigns edges to partitions; a vertex is cut if its adjacent edges are in different partitions. The goal here is to minimize the number of cut vertices while balancing edge counts across partitions. Edge-partitioning-based systems include GraphX^9^. Given the prominent community structure in social networks, algorithms like community detection frequently require accessing adjacent vertex information. Vertex partitioning produces subgraphs that reduce the randomness of memory access for such algorithms, support large-scale parallel processing, and improve computational efficiency. Therefore, this paper employs vertex partitioning for large-scale social network processing.

However, traditional graph partitioning algorithms exhibit intrinsic limitations when applied to social network graphs, particularly due to their disregard for attribute heterogeneity among vertices and edges. This limitation impedes flexible partitioning based on diverse vertex types or partition load. To address this problem, this paper proposes a large-scale social network graph partitioning algorithm called GP-DQN based on DDQN. This method simultaneously ensures load balancing across partitions and minimizes the number of cut edges. The specific works are as follows:

1) Design a partition scoring function to quantify the load and the number of cut edges of partitions. The difference in partition scores before and after a vertex assignment is treated as the reward for that partitioning action.

2) Design a DDQN model that can convolve graph features and perform graph partitioning. Firstly, input the graph feature matrix into a graph convolutional network for processing to obtain a convolutional matrix that integrates vertex attributes, relational structures, and partition load status. This is followed by a linear layer that predicts the partitioning action likely to yield the highest reward.

3) Based on the above two points, the GP-DQN algorithm is proposed, which can ensure partition load balance and minimize the number of cut edges. Experiments demonstrate that the proposed GP-DQN algorithm exhibits excellent partitioning performance on publicly available graph datasets.

## Related work

Applications such as community detection^10^ influence analysis^11^ and link prediction^12^ which are derived from the community structure of social network graphs, all rely on graph partitioning. As a fundamental component of distributed graph processing, graph partitioning assigns large-scale graph data to multiple processors within a computing cluster. Its primary objective is to balance the computational load according to the processing capacity of each processor, while minimizing inter-processor communication to improve overall system performance. To this end, distributed system frameworks such as Spark, Pregel, and Giraph^13^ have been developed successively, providing rich APIs that simplify distributed programming and enable efficient processing of large-scale graphs. Distributed graph computing systems running on homogeneous clusters must ensure load balance across subgraphs and minimize inter-subgraph communication overhead^14^ to achieve high computational efficiency. Extensive research efforts have been devoted to achieving high-performance graph partitioning by scholars worldwide.

In the field of vertex partitioning, the Giraph framework uses a hash function to compute the hash value of each user vertex in the social network and assigns vertices with the same hash value to the same partition for graph partitioning. Kernighan et al.^15^ defined a swap gain function to calculate the cut-edge gain of vertex swaps between partitions, iteratively swapping vertex pairs with the maximum cut-edge gain to generate new partitions. Karypis et al.^16^ designed a multilevel graph partitioning algorithm that iteratively merges tightly connected vertices in the original graph to form a coarsened graph, then refines the merged vertices using the method in paper^15^ to obtain partitions. Wang et al.^17^ proposed a label propagation-based graph partitioning algorithm, updating each vertex’s label to the most common label among its neighbors; when label updates converge, partitions are determined by vertex labels. Cui et al.^18^ combined a genetic algorithm (GA) to treat vertex migration strategies as chromosomes, optimizing partitioning through simulated chromosome crossover and mutation until the optimal strategy is obtained at convergence. Li et al.^19^ developed a dynamic balance algorithm for graph partitioning: first calculating the reduction in cut edges when each vertex migrates to different partitions, transferring the vertex with the maximum reduction to the corresponding partition, adjusting load-imbalanced subgraphs using balance strategies, and employing global memory and perturbation strategies to avoid local optima. Luo et al.^20^ proposed a GN community detection-based graph partitioning algorithm, dividing the graph using non-overlapping community detection after obtaining communities via GN. Graphs with continuously changing data and topological structures are called dynamic graphs. Li et al.^21^ designed a label propagation-based dynamic graph partitioning algorithm for dynamic graphs, using label propagation to identify associated vertex groups of changing vertices, updating affected partitions in real-time via a dynamic processor, and further optimizing partitioning quality with a local optimizer. Nazi et al.^22^ combined graph neural networks (GNNs)^23^ to propose a general graph partitioning framework, using graph convolutional networks (GCNs)^24^ to aggregate vertex feature vectors and connection structures, predicting partitioning strategies via neural networks, and defining a differentiable loss function to compute partitioning loss and improve quality through backpropagation. Gatti et al.^25^ integrated the deep reinforcement learning A2C algorithm^26^ with GNNs to solve the combinatorial optimization problem of graph partitioning: after aggregating vertex features via GCNs, an actor model executes partitioning, while a critic model provides action rewards using normalized cut criteria^27^ to enhance the actor’s partitioning quality.

In the field of edge partitioning, Zhang et al.^28^ proposed a heuristic graph partitioning algorithm focusing on neighbor locality, generating high-locality partitions via neighbor expansion after computing each vertex’s neighbor set. Xie et al.^29^ developed a greedy partitioning algorithm that assigns streaming edges to the endpoint with lower degree, improving partition locality and addressing vertex degree skew effectively. Petroni et al.^30^ proposed a history-based heuristic algorithm, leveraging past partitioning information to compare edge scores across partitions and enhance partitioning quality. Zhao et al.^31^ extended paper^30^ with a heuristic algorithm for social network attributes, maximizing the impact of partitioned edges on memory usage scores of social network attributes and vertex replication scores to improve partitioning quality for attributed social network graphs.

Graph clustering is an unsupervised learning task focusing on graph structures and vertex attributes, aiming to partition vertices into clusters with strongly connected internal structures and highly similar vertex attributes. For instance, Yang et al.^32^ proposed an attribute graph clustering algorithm via approximate generative Bayesian learning, constructing a graph skeleton to retain key information, predicting edge clusters from skeleton edge relationships and vertex attribute distributions, and indirectly deriving vertex clusters. Yang et al.^33^ proposed a graph clustering model combining fuzzy clustering and GCN, predicting vertex-cluster memberships via GCN and multilayer perceptron, optimizing model parameters via a loss function considering intra-cluster structure and attribute similarity, finally labeling vertex clusters by maximum membership. Graph clustering and graph partitioning share highly congruent objectives; thus, some scholars have proposed graph partitioning methods that integrate graph clustering. Mayer et al.^34^ proposed a graph partitioning algorithm based on graph clustering, partitioning vertex clusters by vertex degree, then mapping them to the least loaded partitions in descending order of cluster size, finally partitioning remaining edges via a scoring function considering partition load and cluster size. Ding et al.^35^ proposed a Stackelberg Game-based graph partitioning algorithm, first obtaining head and tail clusters via edge endpoint degrees and information clustering, conducting Stackelberg Game with head clusters as leaders and tail clusters as followers, optimizing cluster-to-partition allocation via a cost function, finally mapping edges to specific partitions.

While the above studies propose methods for load balancing and reducing cross-partition edges from different angles, they have limitations in processing large-scale social networks. Studies^15,16,27^ store the entire graph in memory for partitioning, which risks memory overflow when processing large-scale social networks. Heuristic strategies in paper^17–20,28^ require storing multiple intermediate partitioning results to identify the optimal strategy, leading to high memory usage and excessive computational overhead from frequent result comparisons. Real-time streaming partitioning in paper^29–31^ achieve fast partitioning but yield low-quality results due to their reliance on local graph structures. Studies^22,25^ use GCNs to extract graph structure features and linear networks to predict vertex partitions, but vectorizing vertex and partition features is challenging, making it difficult for GCNs to converge on large-scale social network graphs. Studies^32,33^ focus exclusively on graph structure and attribute information, while overlooking load imbalance caused by cluster size disparities post cluster-to-partition mapping. Studies^34,35^optimizes cluster-to-partition mapping via scoring functions. Its streaming nature reduces memory overhead per traversal and improves partitioning efficiency, yet acquiring high-quality clustering results requires more time. Moreover, such methods neglect attribute heterogeneity of vertices in social networks, potentially leading to excessive load in small-scale clusters. Clustering-based graph partitioning algorithms are more suitable for social networks with clear community structures and homogeneous attributes. In contrast, reinforcement learning-based methods incorporate edge-cut costs and partition loads into reward functions, integrate vertex attributes and partition loads when designing vertex features, and enable flexible partitioning adjustments based on vertex attributes, making them more suitable for social networks with complex attributes. Algorithms in paper^15–20,22,25,27–30^ target non-attributed graphs, while real-world social network graphs have complex vertex and edge attributes; thus, these algorithms cannot adjust partitioning strategies flexibly based on attribute differences, resulting in high cross-partition cut edges and unbalanced loads. Although paper^31^ considers attribute differences, its streaming partitioning fails to capture the full graph structure, leading to poor quality. To address the limitation that existing graph partitioning methods cannot flexibly adapt strategies based on attribute differences in social network graphs, this paper uses GraphSAGE^36^ to convolve vertex attributes, topological structures, and partition load info to represent local vertex-neighbor connections, attribute distributions, and load states. The resulting convolutional matrix is then processed by a DDQN^37^ to get a vertex transfer prediction matrix, from which the optimal graph partition with minimal cut-edges and balanced loads is found.

### Modeling of social network graph partitioning

A social network is a complex structure of individuals and their interrelationships, where entities represent users with distinct identities, and relationships may take the form of friendships, collaborations, communications, information sharing, or other types of interaction.

## Basic concepts

### Definition 1

(*Social Network Attributed Graph*): A social network can be represented as an attributed graph G, a 4-tuple $\:G=\left(V,E,NT,ST\right)$ where the vertex set $\:V=\left\{{v}_{1},{v}_{2},\dots\:,{v}_{n}\right\}$ represents the collection of social network entities with n denoting the number of vertices; the edge set $\:E=\left\{{e}_{1},{e}_{2},\dots\:,{e}_{m}\right\}$ consists of directed edges in the attributed graph with m being the number of directed edges, each edge $\:{e}_{i}=\left({s}_{i},{c}_{i},{et}_{i},{ed}_{i}\right)$ where $\:{s}_{i}$ is the source vertex ID, $\:{c}_{i}$ is the target vertex ID, $\:e{t}_{i}=s{t}_{y}\in\:ST$ denotes the type of edge $\:{e}_{i}$, and $\:{ed}_{i}$ represents the data volume carried by the edge; $\:NT=\left\{n{t}_{1},n{t}_{2},\dots\:,n{t}_{z}\right\}$ is the set of vertex types with z indicating the number of vertex types; $\:ST=\left\{s{t}_{1},s{t}_{2},\dots\:,s{t}_{y}\right\}$ is the set of edge types with y denoting the number of edge types; and each vertex $\:{v}_{i}=\left(i,v{t}_{i},v{d}_{i}\right)$ where *i* is the vertex ID, $\:v{t}_{i}=n{t}_{z}\in\:NT$ represents the type of vertex $\:{v}_{i}$, and $\:v{d}_{i}$ denotes the data volume carried by the vertex.

### Definition 2

(*Social Network Graph Partitioning*) Social network graph partitioning divides the vertices of a graph into multiple mutually exclusive subgraphs. When graph *G* is partitioned into k subgraphs (also called partitions), denoted as $\:P=\left\{{P}_{1},{P}_{2},\dots\:,{P}_{k}\right\}$, it satisfies $\:V={\bigcup\:}_{i=1}^{k}{P}_{i}$ (the union of all partitions equals the vertex set V) and $\:{P}_{i}\cap\:{P}_{j}={\varnothing}$ for any $\:i\ne\:j$(partitions are pairwise disjoint), where $\:{P}_{i}$ represents the $\:i$-th partition of *G*.

If a partition $\:{P}_{i}$ contains a vertex v that has a cut edge with a vertex in another partition, then vertex v is called a boundary vertex of partition $\:{P}_{i}$. If the two vertices of an edge are assigned to different partitions, then the edge is called a cut edge.

### Definition 3

(*The number of cut edges*): The number of cut edges(NCE) can be expressed as the sum of the number of cut edges between different partitions, that is,

1 $$\:NCE\left(P\right)=\frac{1}{2}\sum\:_{i=1}^{k}\sum\:_{j=1}^{k}EC\left({P}_{i},{P}_{j}\right),i\ne\:j$$

Where $\:EC\left({P}_{i},{P}_{j}\right)$ represents the number of cut edges between partition $\:{P}_{i}$ and partition $\:{P}_{j}$.

If graph G is partitioned into k partitions, the load of partition $\:{P}_{i}$, denoted as $\:{ld}_{i}$, is defined as the sum of the data volume carried by all vertices in $\:{P}_{i}$, i.e., $\:{ld}_{i}=\sum\:_{j=1}^{\left|{P}_{i}\right|}\left|{\text{v}\text{d}}_{j}\right|$.

### Definition 4

(*load balance degree*): The load balance degree (LBD) of graph partitioning can be expressed as the difference between the actual partition load and the ideal balanced load, that is,

2 $$\:LBD\left(P\right)=\underset{{P}_{h}\in\:P}{\text{max}}\left|{ld}_{h}-\frac{\sum\:_{i=1}^{k}{ld}_{i}}{k}\right|$$

### Formulaic description of the objective of social network graph partitioning

When vertices in different partitions frequently exchange information—such as during community detection algorithms—limited network bandwidth can degrade data transmission rates, thereby hindering the overall computation process. This phenomenon is termed communication cost, which exhibits a direct positive correlation with the total number of cut edges. To mitigate communication cost, minimizing the count of vertices connected by inter-partition cut edges is essential—specifically, reducing the number of such cross-partition edges. In distributed graph computing, the completion time of computations is governed by the slowest partition’s processing duration. Therefore, when performing graph computations on homogeneous clusters, ensuring load balance across partitions is critical: the graph load allocated to each partition must be as uniform as possible to avoid performance bottlenecks arising from uneven workload distribution. Thus, the objective of social network graph partitioning can be defined as follows:

$$\:min\left[NEC\left(P\right)\right]\:\:\:\:\:\:\:\:\:\:\:\:\:\:\:\:\:\:\:\:\:\:\:\:\:\:\:\:\:\:\:\:\:\:\:\:\:\:\:\:\:\:\:\:\:\:\:\:\:\:\:\:\:\:\:\:\:\:$$

$$\:s.t.\:\:{ld}_{h}\le\:\left(1+\gamma\:\right)\frac{\sum\:_{i=1}^{k}{ld}_{i}}{k},{P}_{h}\in\:P\:\:\:\:\:\:\:\:\:\:\:\:\:\:\:\:\:\:$$

3 $$\:\bigcup\:_{i=1}^{k}{P}_{i}=V,{P}_{i}\bigcap\:{P}_{j}={\varnothing},i\ne\:j$$

In this context, k denotes the number of partitions, and $\:{\upgamma\:}\in\:\left[\text{0,1}\right]$ is the load balance coefficient used to control the upper bound of partition loads. When $\:{\upgamma\:}=0$, it means the load of each partition is identical. When $\:{\upgamma\:}=1$, the upper bound of each partition load is no greater than twice the average partition load; in this case, the algorithm can minimize the number of cut edges under the premise that partition loads do not exceed the upper bound. A higher $\:{\upgamma\:}$ makes it more likely for the algorithm to reduce the number of cut edges, but an excessively high $\:{\upgamma\:}$ can cause severe load imbalance, thereby degrading the performance of graph computation.

### A social network graph partition algorithm based on DDQN

**Fig. 1:**
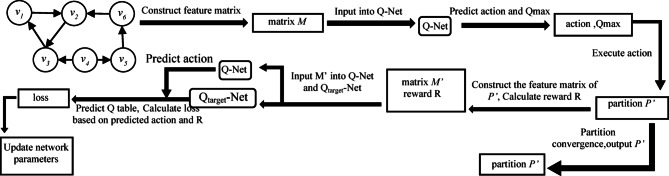
Execution Process of GP-DQN Algorithm.

This paper introduces GP-DQN, a DDQN-based algorithm for social network graph partitioning, designed to minimize cut edges and ensure balanced partition loads. GP-DQN formulates social network graph partitioning as a vertex-centric combinatorial optimization problem in a discrete action space, where each action corresponds to selecting a partition for a given vertex. The algorithm employs GraphSAGE to encode vertex attributes, neighborhood topology, and partition load status, enabling systematic exploitation of both attribute and structural information during partitioning. This approach ensures dual optimization objectives: minimizing inter-partition cut edges and balancing partition load. The GP-DQN workflow is depicted in Fig. 1.

### GraphSAGE and the design of graph vertex feature vectors

GraphSAGE is a graph convolutional network (GCN) employing a hierarchical neighbor sampling strategy. In the sampling stage, it selects a predefined number of neighbors for each vertex to form a sampled neighborhood subset. During convolution, GraphSAGE utilizes an aggregation function to encode vertex attributes, neighborhood connectivity, partition assignment, and partition load profiles within each subset. The output of this aggregation is termed the vertex convolutional feature. In the attribute update stage, vertex attributes in the sampled subset are propagated to incorporate the convolutional features derived from the aggregation step. This sampling-aggregation-update pipeline is repeated across network layers until the predefined depth is reached. The aggregation function of GraphSAGE is as follows:

4 $$\:{h}_{k}\left(v\right)=\sigma\:\left\{{W}_{k}\bullet\:concat\left[{h}_{k-1}\left(v\right),{agg}_{k}\left(v\right)\right]\right\}$$

Where $\:{h}_{k}\left(v\right)$ is the feature embedding of the *k*-th layer *v*, $\:{W}_{k}$ is the weight matrix, and $\:\sigma\:$ is the activation function,$\:\:{agg}_{k}\left(v\right)$ is the mean of the neighborhood features of *v*. GraphSAGE’s local sampling strategy and multi-layer aggregation method enable higher efficiency in capturing local structures and hierarchical relationships of social networks, making it suitable for fast convolution of vertex features in large-scale social network graphs.

Social network graphs are characterized by complex vertex attributes, heavy-tailed degree distributions, high clustering coefficients, and pronounced community structures. Given this attribute complexity, balancing only the vertex count across partitions during iterative graph partitioning induces excessive load discrepancies. This imbalance thereby increases inter-partition cut edges, diverting substantial computational resources to inter-partition communication overhead and partition synchronization delays.

This paper encodes vertex-related information—comprising vertex attributes, neighborhood connectivity, partition assignments, and partition load profiles—as input features for GraphSAGE to perform hierarchical convolution on social network graphs. The encoded features explicitly model vertex attributes, topological adjacency, and partition-specific load states, enabling the algorithm to dynamically capture inter-vertex relational dependencies. This approach enhances the model’s capacity for feature extraction and generalization in graph partitioning scenarios. The specific implementation process is as follows:

Firstly, obtaining the K-hop subgraph $\:{G}_{sub}$ of the boundary vertex set, then construct an attribute feature vector $\:F\left(v,P\right)$ for each vertex v in the subgraph $\:{G}_{sub}$, which specifically includes the following feature information:

1)The data volume of v, denoted as $$\:Data\left(v\right)=\raisebox{1ex}{${vd}_{v}$}\!\left/\:\!\raisebox{-1ex}{$\underset{{u\epsilon V}}{\text{max}}{vd}_{u}$}\right.$$. This feature value is a normalized calculation of the data volume carried by verte v, representing the measured value of the vertex data volume feature dimension in $$\:F\left(v,P\right)$$.

2)The type of v, denoted as $\:Type\left(v\right)=OneHot\left({vt}_{v}\right)$. This feature value is the one-hot encoding of vertex v’s type, representing the encoded value of the vertex type feature dimension in $\:F\left(v,P\right)$.

3)The partition location of v, denoted as $\:partition\left(v\right)=OneHot\left(i\right)$ such that $\:v \epsilon {P}_{i}$. This feature value is the one-hot encoding of the partition number where vertex v is located, representing the encoded value of the partition location feature dimension in $\:F\left(v,P\right)$.

4)The load status of partition P, denoted as $$\:Load\left(P\right)$$. This feature value is a list with a length equal to the number of partitions, where the j-th element is the ratio of the load of partition j to the total load, i.e., $$\:\raisebox{1ex}{${ld}_{j}$}\!\left/\:\!\raisebox{-1ex}{$\sum\:_{i=1}^{k}{ld}_{i}$}\right.$$, representing the current load status of partitions in $$\:F\left(v,P\right)$$.

Immediately afterwards, combine and flatten the above-mentioned information to obtain the feature vector $\:F\left(v,P\right)$ of vertex v. Then, combine the feature vectors of each vertex $\:{v}_{i}$ in $\:{G}_{sub}$ into a feature matrix:

5 $$\:M=\left[\begin{array}{c}\begin{array}{c}F\left({v}_{1},P\right)\\\:F\left({v}_{2},P\right)\end{array}\\\:\dots\:\\\:F\left({v}_{{n}^{{\prime\:}}},P\right)\end{array}\right]$$

Then, represent the vertex connection relationships of $\:{G}_{sub}$ as a matrix EG with 2 rows and the number of columns equal to the number of edges in $\:{G}_{sub}$. In the first row, store the starting points, and in the second row, store the ending points. Two values in the same column represent the two endpoints of an edge. Taking the matrix EG and feature matrix M as inputs to the GraphSAGE layer in DDQN, after several layers of graph convolution, the resulting graph convolution output is a high-dimensional feature matrix that aggregates vertex attribute information, partition load status, and the connection structure of $\:{G}_{sub}$. Through this matrix, the local structural information of each vertex in $\:{G}_{sub}$ can be captured, enhancing both the representation of partition loads and the feature representations of individual vertices. This provides the fully connected layer with rich information about the vertices, edges, and partitions of $\:{G}_{sub}$, thereby improving the prediction accuracy of the Q-value matrix.

### DDQN and the design of graph partitioning actions

DDQN is an advanced deep reinforcement learning (DRL) algorithm tailored to address the graph partitioning problem. Its primary network, Q-Net, functions to select the action $\:a$ with the maximum Q-value in the current state $\:s$—where actions represent vertex-partition assignment decisions—and the action $\:{a}^{{\prime\:}}$ with the maximum Q-value in the subsequent state $\:{s}^{{\prime\:}}$. The target network, Qtarget-Net, evaluates the Q-value of executing $\:{a}^{{\prime\:}}$ in $\:{s}^{{\prime\:}}$ post-partitioning. Through iterative parameter updates between Q-Net and Qtarget-Net, DDQN learns to select actions that maximize cumulative rewards, ultimately converging on an optimal graph partitioning strategy. DDQN exhibits high adaptability in partitioning large-scale social network graphs, for the following reasons: First, DDQN decouples action selection from action evaluation during training, which reduces the likelihood of overestimating Q-values for partitioning actions—a common issue in the original DQN—thus improving both partitioning quality and training efficiency. Second, since social network graph partitioning is inherently a vertex-level combinatorial optimization problem, DDQN’s discrete action space allows it to directly predict Q-values for specific vertex-partition pairs, thereby tightly linking partitioning objectives with action choices. Finally, by learning and leveraging the complex attributes, topological structures, cut costs, and load distributions of large-scale social networks, DDQN optimizes long-term cumulative rewards and continually refines its graph partitioning strategy.

Formally, a state encapsulates all environment-relevant information at a given time step; an action denotes the agent’s partition assignment decision in that state; and a reward represents the scalar feedback received upon executing that action. In this context, the objective of DRL is to maximize the cumulative reward—defined as the expected return over state-action pairs under fixed network parameters—through policy optimization. In GP-DQN, the state comprises the vertex feature matrix and graph topology, while the action corresponds to transferring vertices between partitions.

After GraphSAGE convolves vertex features and topology, the resulting feature matrix is passed through Q-Net’s linear layer to predict the Q-value matrix. Next, the maximum Q-value ($\:{Q}_{max}$) and its corresponding target vertex-partition assignment are identified, followed by reassigning the target vertex to the chosen partition. After reassignment, a reward—calculated from load balance and the number of cut edges—is obtained. The target Q-value is then computed by combining this reward with the Qtarget-Net’s evaluation of the action. Finally, the loss is calculated as the difference between the target Q-value ($\:{Q}_{target}$) and $\:{Q}_{max}$. This loss guides updates to the parameters of both Q-Net and Qtarget-Net, enabling Q-Net to consistently select actions that maximize cumulative rewards, thereby determining the optimal partitioning policy.

Although deeper neural networks generally enhance generalization, feature learning, and representation capabilities, excessive depth can cause issues such as over-smoothing, overfitting, and exponential increases in computational cost and latency. To balance representational power and avoid overfitting, this paper adopts a pragmatic DDQN architecture: a three-layer graph convolutional network (GCN) to learn vertex features and topology, followed by two fully connected layers that map GCN features to the action space.

**Fig. 2:**
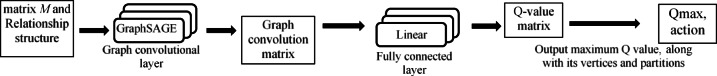
The structures of the Q network and the target Q network.

ReLU activation functions are applied between each layer in the DDQN network to introduce non-linearity. Q-Net and Qtarget-Net share the same network architecture, detailed in Fig. 2.

In the i-th partitioning step, the vertex feature information and relationship structure of $\:{G}_{sub}$ are used by Q-Net to predict a Q-value matrix (Q-table) of size $\:{n}^{{\prime\:}}$ $\:\times\:$ k (where $\:{n}^{{\prime\:}}$ is the number of vertices in $\:{G}_{sub}$ and k is the number of partitions), representing the expected Q-value when a vertex is transferred to a specific partition. The horizontal axis of the Q-table denotes the target partition index, and the vertical axis denotes the target vertex index, mapping the problem of selecting target vertices and partitions in graph partitioning to the discrete action space of DDQN. The Q-value matrix $\:{QM}_{i}$ is defined as:

6 $$\:{QM}_{i}=\left[\begin{array}{cccc}{Q}_{11}&\:{Q}_{12}&\:\dots\:&\:{Q}_{1k}\\\:{Q}_{21}&\:{Q}_{22}&\:\dots\:&\:{Q}_{2k}\\\:\dots\:&\:\dots\:&\:\dots\:&\:\dots\:\\\:{Q}_{{n}^{{\prime\:}}1}&\:{Q}_{{n}^{{\prime\:}}2}&\:\dots\:&\:{Q}_{{n}^{{\prime\:}}k}\end{array}\right]$$

Where $\:{Q}_{xy}$ represents the expected Q-value when the x-th vertex in $\:{G}_{sub}$ is transferred to partition y. Next, the maximum value $\:{Q}_{max}$ in $\:{QM}_{i}$ and its corresponding target vertex index and target partition index are identified. Finally, in partition $\:{P}_{i-1}$, transferring the target vertex to the target partition yields the partition result $\:{P}_{i}$ and the reward $\:{Reward}_{i}$ for this partitioning action. If the expected Q-value of transferring the target vertex to its current partition is $\:{Q}_{max}$, no transfer is performed, and the reward $\:{Reward}_{i}$ for this action is set to 0.

## Design of graph partitioning action reward

This paper employs a partition scoring function that computes a weighted sum of the normalized partition load balance and the number of cut edges. A higher score indicates either a larger number of cut edges or greater imbalance in partition loads, both of which degrade graph computation performance due to increased inter-partition communication and synchronization overhead. If a partition fails to meet the load balance requirement, the current partitioning is considered a failure, and the score is set to the maximum value of 1. The partition scoring function $\:Score\left(P\right)$ is defined as follows:

7 $$\:Score\left(P\right)=\left\{\begin{array}{c}1,if\:{ld}_{h}>\left(1+\gamma\:\right)\frac{\sum\:_{i=1}^{k}{ld}_{i}}{k},{P}_{h}\in\:P\\\:\mu\:\bullet\:\frac{\sum\:_{i=1}^{k}\sum\:_{j=1}^{k}EC\left({P}_{i},{P}_{j}\right)}{2\left|E\right|}+\\\:\left(1-\mu\:\right)\frac{\underset{{P}_{h}\in\:P}{\text{max}}\left|{ld}_{h}-\frac{\sum\:_{i=1}^{k}{ld}_{i}}{k}\right|}{\sum\:_{i=1}^{k}{ld}_{i}},otherwise\end{array}\right.$$

Where $\:\gamma\:$ is the load balance coefficient, and $\:\mu\:\in\:\left[\text{0,1}\right]$ is the reward balance coefficient. When $\:\mu\:=0$, the system only considers partition load balance without cut edges; when $\:\mu\:=1$, it only considers cut edges without load balance. After a partitioning action is completed, the reward for the action is the difference in scores between the two partition sets before and after partitioning. A positive reward indicates that the partitioning action helps achieve partitions with the minimum number of cut edges and balanced loads. Reward for the partitioning action:

8 $$\:{\text{R}\text{e}\text{w}\text{a}\text{r}\text{d}}_{i}=\text{S}\text{c}\text{o}\text{r}\text{e}\left({P}_{i-1}\right)-\text{S}\text{c}\text{o}\text{r}\text{e}\left({P}_{i}\right),i>0$$

Where $\:{\text{R}\text{e}\text{w}\text{a}\text{r}\text{d}}_{i}$ represents the reward value obtained from the i-th partitioning. When i = 1, $\:\text{S}\text{c}\text{o}\text{r}\text{e}\left({P}_{0}\right)$ represents the score of the partition $\:{P}_{0}$ after the initial partitioning.

### Design of DDQN parameter update mechanism

In DDQN, the Q-Net is responsible for selecting the graph partitioning action $\:a$ in the current state $\:s$ and the graph partitioning action $\:{a}^{{\prime\:}}$ in the next state $\:{s}^{{\prime\:}}$. The Qtarget-Net is responsible for evaluating the Q-value of executing $\:{a}^{{\prime\:}}$ in $\:{s}^{{\prime\:}}$. After DDQN completes the i-th graph partitioning and obtains the K-hop subgraph $\:{G}_{sub}^{{\prime\:}}$ of the new boundary vertex set, the Qtarget-Net is used to evaluate $\:{G}_{sub}^{{\prime\:}}$ and obtain $\:{QM}_{i}^{{\prime\:}}$. The Q-Net predicts $\:{G}_{sub}^{{\prime\:}}$ to get the target vertex $\:{x}^{{\prime\:}}$ and the target partition $\:{y}^{{\prime\:}}$ corresponding to the maximum Q-value, and $\:{QM}_{i}^{{\prime\:}}[{x}^{{\prime\:}},{y}^{{\prime\:}}]$ is taken as the evaluated Q-value of $\:{G}_{sub}^{{\prime\:}}$. The target Q-value $\:{Q}_{target}$ of this action is calculated according to the reward value of the i-th partitioning action and the evaluated Q-value of $\:{G}_{sub}^{{\prime\:}}$:

9 $$\:{Q}_{target}={Reward}_{i}+{\upalpha\:}\bullet\:{QM}_{i}^{{\prime\:}}\left[{x}^{{\prime\:}},{y}^{{\prime\:}}\right]$$

Where $\:{\upalpha\:}$ is used to balance the emphasis on immediate rewards and future rewards. When $\:{\upalpha\:}$ is small, the model tends to immediate rewards; when $\:{\upalpha\:}$ is large, the model tends to future rewards. When measuring the difference between $\:{Q}_{target}$ and $\:{Q}_{max}$, to improve the model’s sensitivity to abnormal expected Q-values, this paper uses the mean-squared error loss function to calculate the loss value between $\:{Q}_{target}$ and $\:{Q}_{max}$. Finally, the parameters of the Q-Net model are updated through the gradient descent algorithm. Initially, the Q-Net and the Qtarget-Net have the same structure and the same initial parameters. After several updates of the Q-Net model parameters, the parameters of the Q-Net model are assigned to the Q_target_-Net model.

Finally, this paper proposes the GP-DQN algorithm (as detailed in Algorithm 1) to address the large-scale social network graph partitioning problem, minimizing the number of cut edges while ensuring load balance. The algorithm takes as input the social network graph, model scoring parameters, the number of subgraph hop, Qtarget-Net update intervals, and load balance coefficient. The output is the partition set.

**Algorithm 1:**
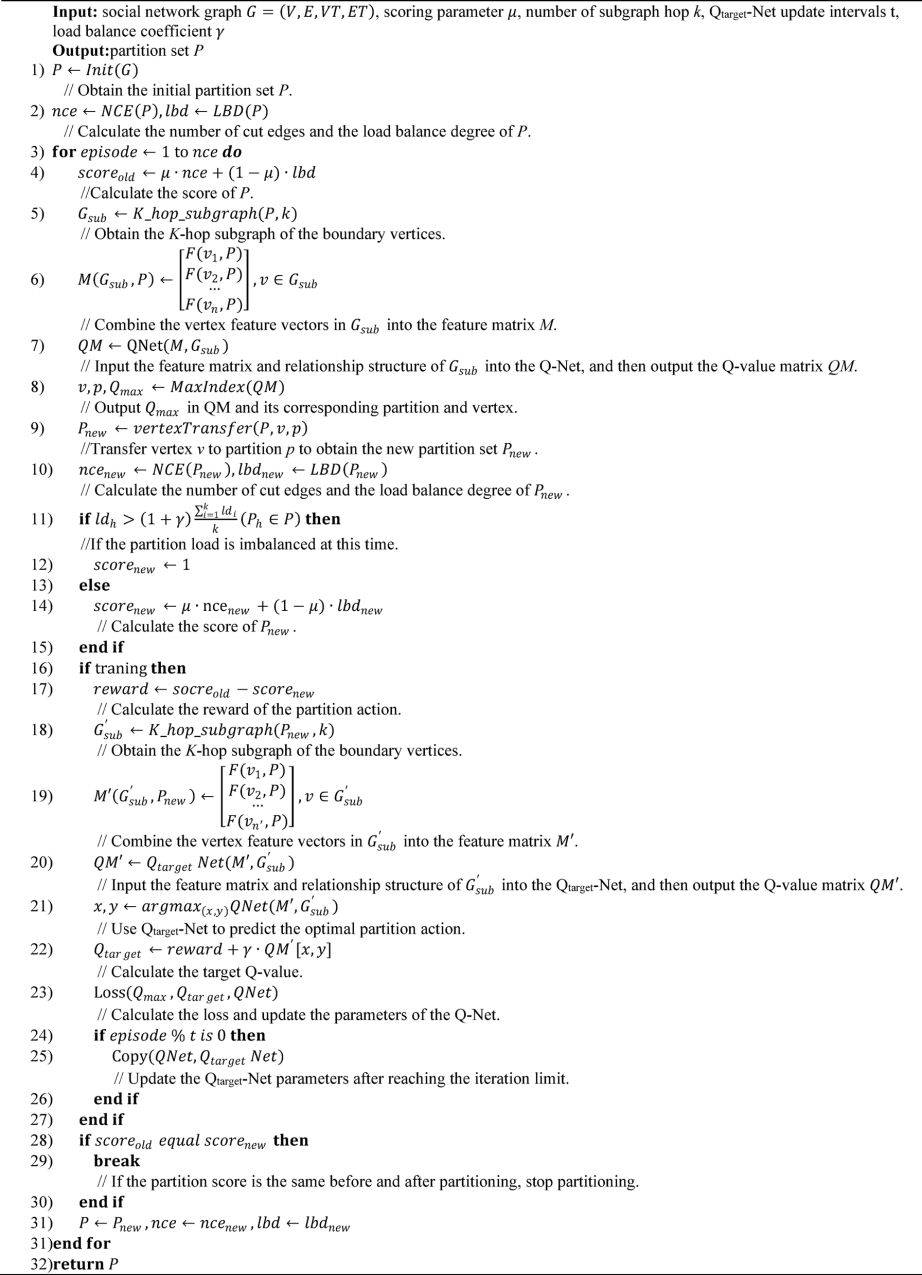
GP-DQN.

## Results and discussion

To evaluate the performance of GP-DQN in partitioning social network graphs, four sets of experiments were conducted for validation.

### Experimental environment and dataset

This paper uses five social network graph datasets and one web network graph dataset from Stanford University to evaluate the performance of the proposed GP-DQN algorithm compared with DRL-Metis, Metis, and Hash in partitioning different graphs. The six datasets are shown in Table 1.

**Table 1:** Social network graph datasets.

Dataset	Number of Vertices	Number of Edges	Average Degree of Vertices	Type
FaceBook	22,470	171,002	15.22	Social network
Amazon	334,863	925,872	5.53	Web network
NotreDame	325,729	1,090,108	6.69	Social network
LastFM	7,624	27,806	7.29	Social network
Twitch	7,126	35,324	9.91	Social network
GitHub	37,700	289,003	15.33	Social network

As shown in Table 1, the Facebook and GitHub datasets have higher average degrees; the Amazon and NotreDame datasets contain more vertices and edges; while the LastFM and Twitch datasets have fewer vertices and edges but higher average degrees than Amazon and NotreDame. Hash, Metis, and DRL-Metis are selected as comparison algorithms for the following reasons: (1) Hash, the simplest partitioning algorithm, is commonly used as the default in many distributed systems; (2) Metis, a widely used algorithm, achieves high performance through its distinctive hierarchical mechanism; (3) DRL-Metis, like GP-DQN, is a reinforcement learning-based partitioning algorithm, differing in its use of A2C whereas GP-DQN employs DDQN.

The experimental setup consists of two identically configured servers connected via optical fiber. Each server is equipped with an Intel(R) Core(TM) i7-9500 H CPU running at 2.60 GHz, 16 GB of RAM, a 1 TB mechanical hard drive, and runs the CentOS 7 operating system.

### Comparative experiments of load balance degree and number of cut edges

**Fig. 3:**
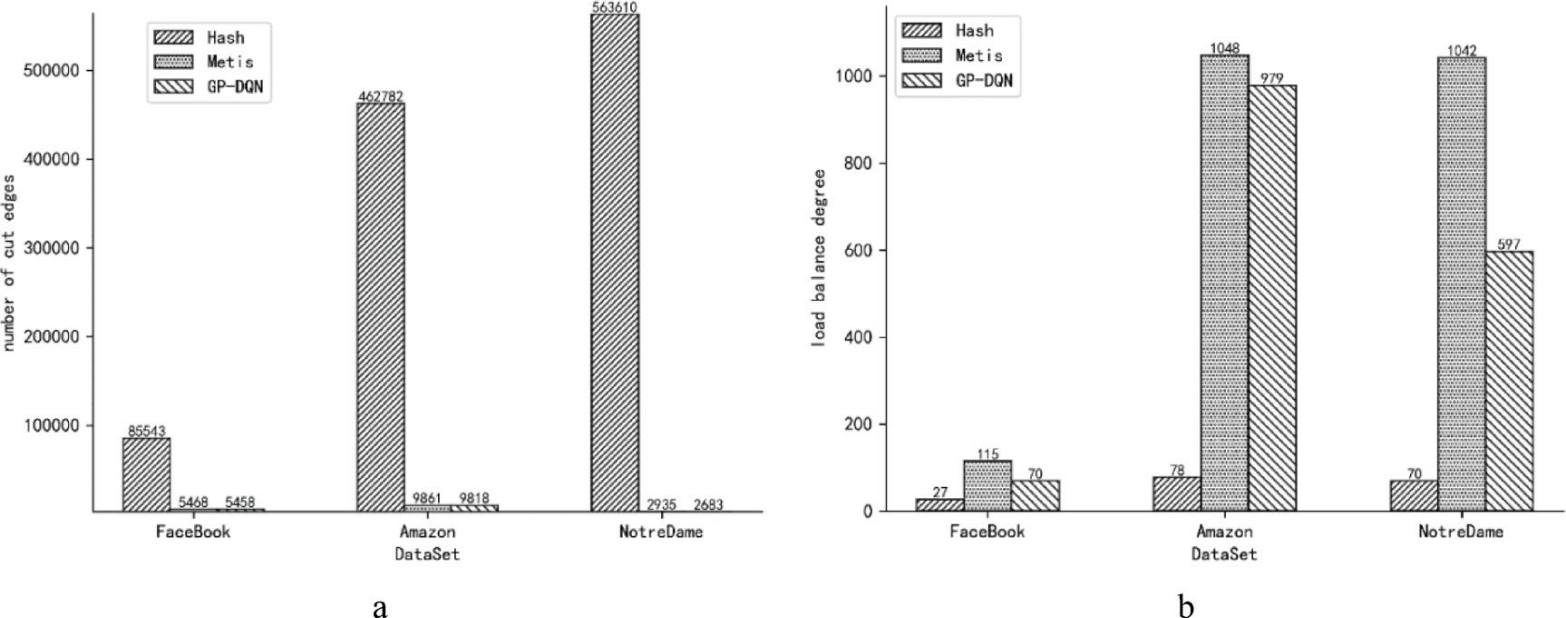
(**a**) The number of cut edges of the three algorithms on different datasets. (**b**) The load balance degree of the three algorithms on different datasets.

This experiment evaluates the differences in load balance degree and number of cut edges among GP-DQN, Metis, and Hash on three datasets: Facebook, Amazon, and NotreDame, using a single server. The load balance coefficient is set to 0.03, meaning that if the load of any partition deviates by more than 3% from the average partition load, it is considered unbalanced. The partition reward balance coefficient µ is set to 0.5. The load balance degrees and numbers of cut edges for the three algorithms across different datasets are presented in Fig. 3a, b.

As shown in Fig. 3a, b, Hash exhibits the lowest load balance degree and the highest number of cut edges. Running graph computations on Hash partitions can cause severe communication delays due to the excessively high number of cut edges, leading to significant overall computation time spent waiting for inter-partition communication. Metis achieves the highest load balance degree, which can result in resource underutilization when processing partitions with relatively low loads. GP-DQN achieves the fewest cut edges, with a load balance degree between that of Hash and Metis. This balance results in moderate communication delays when processing partitions across two servers while ensuring efficient utilization of server resources, thereby attaining the fastest overall computation time.

These differences in partitioning outcomes arise because Hash employs a vertex-uniform partitioning strategy, which results in the smallest load balance degree but neglects the influence of graph topology on partition quality, thereby producing a large number of cut edges across partitions. Both Metis and GP-DQN consider cut edges and load balance; however, Metis balances partitions primarily by vertex count without accounting for vertex attribute differences, leading to small vertex count disparities but significant actual load imbalances. In GP-DQN, GraphSAGE encodes graph topology and vertex attribute differences to extract features representing vertex attributes, neighborhood structures, and partition loads. DDQN integrates load balance and cut edge considerations into its model and autonomously optimizes partitioning actions, enabling dynamic adjustment of partition loads based on vertex data differences, thereby further reducing load imbalance and cut edges. Moreover, as the Amazon graph dataset is a Web graph characterized by weak community structure in both attributes and topology, GP-DQN cannot fully exploit attribute differences and neighborhood information to flexibly adjust partitions, resulting in relatively higher load imbalance in the partitioning of the Amazon dataset.

### Comparative experiments on the time of running graph computations on different partitioning results

**Fig. 4:**
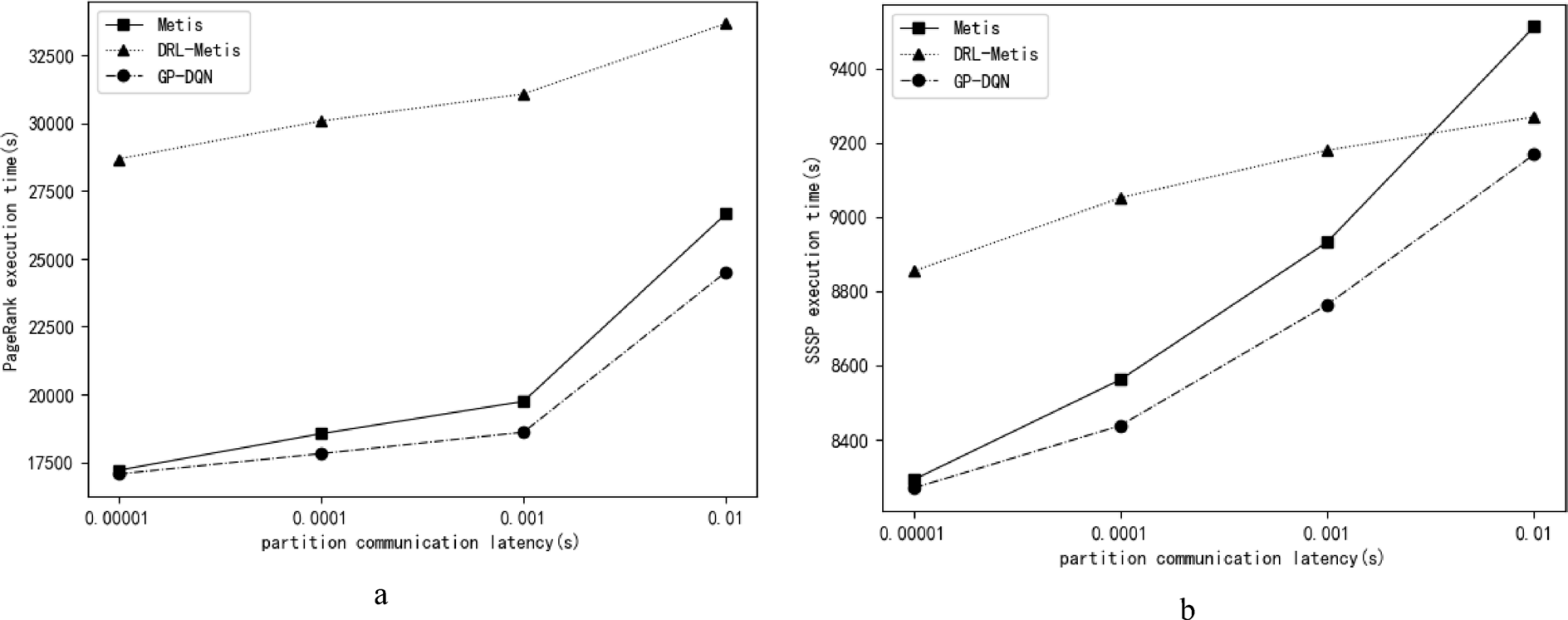
(**a**) Running Time of PageRank. (**b**) Running Time of SSSP.

After GP-DQN, DRL-Metis, and Metis partition the Facebook dataset, each algorithm produces two partitions that are assigned to two servers respectively. The PageRank and Single-Source Shortest Path (SSSP) algorithms are executed on these partitions, and their running times are compared. The running times of PageRank and SSSP increase as the partition communication delay—the time required for a message to travel between partitions along topological edges—increases. We compare the running times of PageRank and SSSP on partitions generated by the three algorithms under four partition communication delays: 0.00001 s, 0.0001 s, 0.001 s, and 0.01 s. In the experiment, PageRank is run for 100 iterations, and each vertex in the graph serves as the source for SSSP once. The load balance coefficient is set to 0.03. The reward balance coefficient µ is set to 0.2, 0.4, 0.6, and 0.8 corresponding to partition communication delays of 0.00001 s, 0.0001 s, 0.001 s, and 0.01 s, respectively. The running times of PageRank and SSSP on partitions generated by the three algorithms under different partition communication delays are shown in Fig. 4a, b.

As can be seen from Fig. 4a, b, the running times of both PageRank and SSSP are the shortest on the partitions obtained by GP-DQN. PageRank runs slowest on the partitions of DRL-Metis. Within the partition communication delay range of 0.00001s to 0.001s, SSSP runs slowest on DRL-Metis partitions, while at a delay of 0.01s, SSSP runs slowest on Metis partitions. Specifically, the execution time differences of PageRank and SSSP on the respective partitioning results of GP-DQN, Metis, and DRL-Metis under varying partition communication delays are as follows: at 0.00001s delay, PageRank runtime on GP-DQN is 13,500s faster than Metis and 1,161,200s faster than DRL-Metis, while SSSP runtime is 22.61s faster than Metis and 584.35s faster than DRL-Metis; at 0.0001s delay, PageRank on GP-DQN outperforms Metis by 73,500s and DRL-Metis by 1,226,300s, with SSSP runtime 123.59s faster than Metis and 613.44s faster than DRL-Metis; at 0.001s delay, GP-DQN’s PageRank runtime is 113,600s faster than Metis and 1,247,200s faster than DRL-Metis, and SSSP runtime is 168.65s faster than Metis and 415.83s faster than DRL-Metis; at 0.01s delay, PageRank on GP-DQN is 214,500s faster than Metis and 915,900s faster than DRL-Metis, while SSSP runtime is 343.92582s faster than Metis and 101.24982s faster than DRL-Metis.

These results arise because PageRank requires frequent iterations to update each vertex’s PageRank value. Thus, the slowdown in iteration speed due to partition overload has a greater impact on PageRank’s overall computation time than the increased communication delay caused by a high number of cut edges. Consequently, load balance influences PageRank’s computation speed more significantly than the number of cut edges. The DRL-Metis algorithm’s reward function prioritizes minimizing cut edges and in-partition degrees, which often results in unbalanced partition loads and consequently the slowest PageRank performance. The SSSP algorithm requires more frequent cross-partition neighbor information access and depends more heavily on minimizing cut edges compared to PageRank. At higher partition communication delays, DRL-Metis partitions have the fewest cut edges, resulting in SSSP running times that are only exceeded by those of GP-DQN. Metis balances vertex counts and cut edges during partitioning. For communication delays between 0.00001 s and 0.001 s, PageRank and SSSP running times on Metis partitions are significantly lower than those on DRL-Metis but slightly higher than on GP-DQN. However, Metis does not account for vertex data volume or communication delays, limiting its ability to adjust partition loads flexibly based on load and delay constraints. Consequently, it exhibits the slowest SSSP performance at a 0.01 s communication delay. GP-DQN adapts to varying partition communication delays by tuning the reward balance coefficient, achieving the fastest execution times for both PageRank and SSSP across all tested delay scenarios.

### Comparative experiments on partitioning time of different graph datasets

**Fig. 5:**
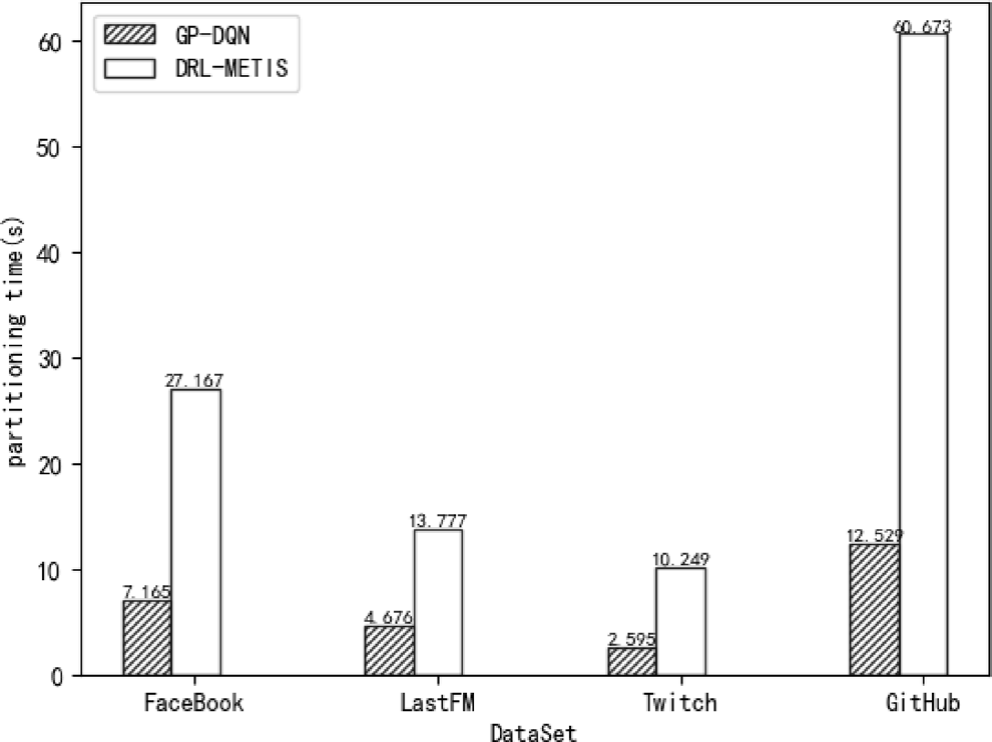
Partitioning time of GP-DQN and DRL-Metis on different datasets.

In this experiment, GP-DQN and DRL-Metis partition four graph datasets—Facebook, LastFM, Twitch, and GitHub—on a single server, and their partitioning times are compared. The load balance coefficient is set at 0.03, and the reward balance coefficient µ is fixed at 0.5. Figure 5 illustrates the partitioning times of GP-DQN and DRL-Metis across the four datasets.

As can be seen from Fig. 5, GP-DQN partitions graphs faster than DRL-Metis on all four datasets. Specifically, GP-DQN reduces partitioning time compared to DRL-Metis by 20.00 s for Facebook, 9.10 s for LastFM, 7.65 s for Twitch, and 48.14 s for GitHub. This is because DRL-Metis employs policy gradients to generate continuous action probabilities from states, selects the highest-probability action, and calculates rewards to update network parameters. However, graph partitioning is a discrete action problem involving vertex-to-partition assignments, and DRL-Metis—better suited for continuous action spaces—performs poorly in such discrete scenarios. In contrast, GP-DQN’s discrete action selection aligns naturally with the combinatorial optimization nature of graph partitioning, leading to significantly faster partitioning times.

### Adaptability analysis of GP-DQN to load balance and communication delay with different reward balance coefficients

**Fig. 6:**
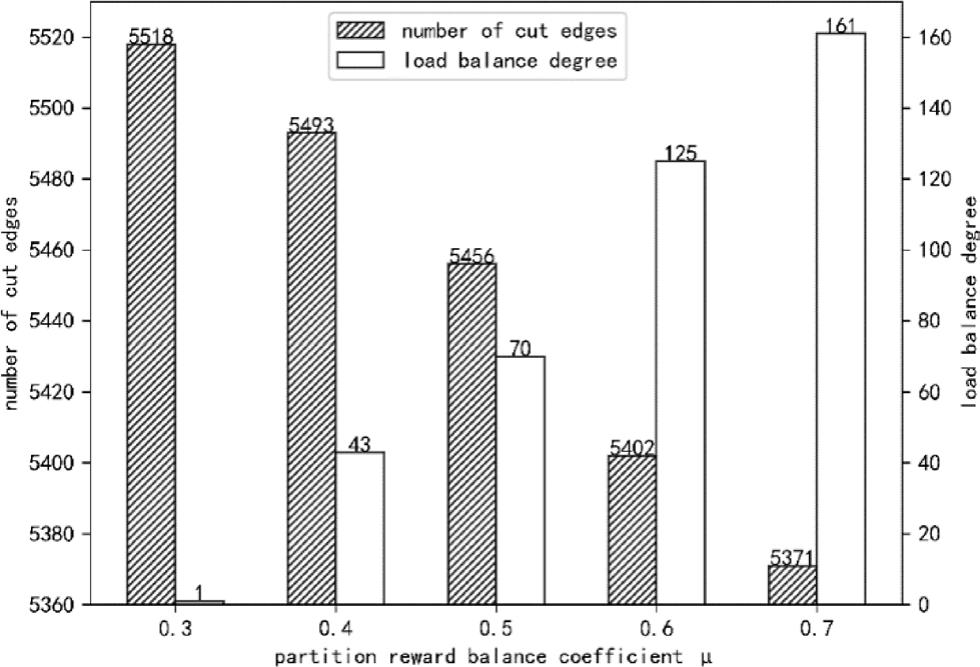
Load balance degree and number of cut edges of GP-DQN under different reward balance coefficients.

In this experiment, GP-DQN partitions the Facebook dataset on a single server using varying reward balance coefficients. The algorithm’s adaptability to varying partition communication delays and load balance coefficients is evaluated by examining the load balance degree and the number of cut edges in the resulting partitions. Figure 6 presents the load balance degrees and numbers of cut edges for GP-DQN partitions obtained with different reward balance coefficients.

Figure 6 shows that at µ = 0.3, GP-DQN produces the highest number of cut edges and the lowest load balance degree, making it more suitable for scenarios with low load balance coefficients and low partition communication delays. At µ = 0.7, the algorithm attains the lowest number of cut edges and the highest load balance degree, making it better suited for environments with high load balance coefficients and high partition communication delays. At µ = 0.5, GP-DQN achieves a balanced trade-off between cut edges and load balance, making it appropriate for scenarios with low load balance coefficients but high partition communication delays. All five reward balance coefficients tested in this experiment correspond to scenarios characterized by high load balance requirements and low partition communication delays.

## Summary

This paper proposes GP-DQN, a DDQN-based algorithm to address the large-scale social network graph partitioning problem. It employs GraphSAGE to encode vertex attribute features and neighborhood structures, then feeds the resulting representations into DDQN for forward propagation to identify partitioning actions that minimize cut edges and balance partition loads. However, GP-DQN has several limitations: first, it is designed for static social network graphs and performs poorly on dynamic graphs; second, it targets homogeneous cluster environments and lacks adaptability to heterogeneous clusters. Future work will focus on two directions: first, developing a more time-efficient graph partitioning algorithm for dynamic graphs that meets real-time computation demands while maintaining partitioning quality; second, designing a graph partitioning algorithm capable of accommodating cluster heterogeneity by dynamically adjusting partitions according to variations in cluster communication and performance. Experimental results demonstrate that GP-DQN produces load-balanced graph partitions with fewer cut edges, resulting in faster graph computation within each partition.

## Data Availability

No new data were generated in this study. The analyses utilized publicly available datasets from the Stanford Network Analysis Project (https://snap.stanford.edu/).
